# Esophageal variceal ligation plus sclerotherapy vs. ligation alone for the treatment of esophageal varices

**DOI:** 10.3389/fsurg.2022.928873

**Published:** 2022-10-14

**Authors:** Xiaofen Yue, Zeyu Wang, Jianbiao Li, Xiaoling Guo, Xiehua Zhang, Shengnan Li, Hongcheng Lv, Dongsheng Hu, Xiangjun Ji, Shuang Li, Wei Lu

**Affiliations:** ^1^ Department of Hepatobiliary Oncology, Liver Cancer Center, Tianjin Medical University Cancer Institute and Hospital, National Clinical Research Center for Cancer, Key Laboratory of Cancer Prevention and Therapy, Tianjin’s Clinical Research Center for Cancer, Tianjin Medical University, Tianjin, China; ^2^ Department of Hepatology, Tianjin Second People’s Hospital, Tianjin Institute of Hepatology, Tianjin, China; ^3^ Department of Clinical Laboratory, The Third Central Hospital of Tianjin, Tianjin Key Laboratory of Extracorporeal Life Support for Critical Diseases, Artificial Cell Engineering Technology Research Center, Tianjin, China; ^4^ Department of Infectious Diseases, The First Affiliated Hospital of Baotou Medical College, Baotou, China; ^5^ Department of Oncology, Tianjin Academy of Traditional Chinese Medicine Affiliated Hospital, Tianjin, China

**Keywords:** esophageal variceal bleeding, esophageal variceal ligation, endoscopic injection sclerosis, rebleed, recurrence

## Abstract

**Background:**

This study aimed to evaluate the efficacy and adverse events of esophageal variceal ligation (EVL) vs. EVL combined with endoscopic injection sclerosis (EIS) in the therapy of esophageal varices.

**Methods:**

Patients from January 2017 to August 2021 who received EVL alone (control group) or EVL plus EIS (intervention group) were enrolled in this retrospective study. Efficacy, including rebleeding (clinically hematemesis or melena, confirmed by endoscopy as esophagogastric varices bleeding), variceal recurrence rate (the presence of esophagogastric varices which is needed to be treated again) the number of sessions performed to complete eradication of varices, and safety (adverse events) were compared. The variceal recurrence-associated factors were derived by univariate and multivariate logistic regression analyses.

**Results:**

The variceal recurrence and rebleeding rate in the intervention group showed significantly lower than the control group (2.6% vs 10.3%, *P* = 0.006 and 20.7% vs 37.5%, *P* = 0.029, *P* = 0.006, respectively, in the 12-month follow-up). The adverse events (fever, chest pain, swallowing, and esophageal stricture) showed no significant difference between the two groups (*P *> 0.05). Further research showed that the efficacy of the intervention group was better than the control group only achieved in prophylactically endoscopic treatment patients. The diameter of esophageal varices and gastric varices co-exist showed significant effects on variceal recurrence in intervention group [odds ratio (OR) = 15.856; 95% confidence interval (CI), 1.709–160.143; *P* = 0.016 and OR = 4.5; 95% CI, 1.42–20.028; *P* = 0.021; respectively].

**Conclusions:**

The intervention group may obtain lower recurrence, rebleeding rate, and fewer sessions performed to complete eradication of varices (number of sessions) and similar incidence of adverse events, especially for prophylactically treatment. Among the intervention group, the diameter of esophageal varices and gastric varices were closely associated with variceal recurrence.

## Introduction

Esophageal variceal bleeding (EVB) is one of the most severe adverse events of liver cirrhosis (1). Seven percent of patients with liver cirrhosis develop the symptom of esophagogastric fundic varices each year (2). Patients with cirrhosis who are not treated prophylactically have a high risk of rebleeding within 1 year and a mortality rate of approximately 15%–20% within 6 weeks (3). Current guidelines recommend carvedilol, non-selective beta-blockers (NSBBs), or variceal band ligation for primary prevention of esophageal variceal (4). NSBBs are not indicated in patients with low basal heart rates or refractory ascites, and they cannot be evaluated for their ability to reduce portal vein pressure. Esophageal variceal ligation (EVL) is considered to have a high variceal recurrence rate since EVL only ligates varices, achieving local eradication around the ligature site, with no effect on the connecting perforating vessels or varices (5, 6). In contrast, post-EVL ulcers bleed in 5%–10% of cases within 3–7 days after surgery, with a mortality rate of 28% (7). However, endoscopic injection sclerosis (EIS) can obliterate the interconnected perforating vessels and veins that feed varicose (6, 8). Moreover, due to extensive wall necrosis, its dangerous post-sclerotherapy adverse events are caused mainly by incorrect injection techniques, over-injection of sclerosing agents, or the use of high concentrations of sclerosing agents (9).

In recent years, some evidence suggests that EVL combined with EIS can significantly reduce bleeding and recurrence of esophageal varices (10, 11), with similar adverse events compared to EVL alone. Some studies suggest that the addition of sclerotherapy to endoscopic band ligation has not changed the clinically relevant outcomes (variceal rebleeding, death, time to variceal obliteration) (12, 13). However, the samples of most of these studies were small. Therefore, we established a large-scale retrospective study to investigate the advantages and disadvantages of EVL and EVL plus EIS for esophageal varices. Moreover, further screened the patients suitable for EVL plus EIS.

## Materials and methods

### Patients' clinical and laboratory data collection

Three hundred and ten patients who received EVL (control group, *n* = 136) or EVL plus EIS (intervention group, *n* = 174) at Tianjin Second People's Hospital between January 2017 and August 2021 were included in this retrospective study. Patients included in the study were those with cirrhosis and esophageal varices with or without gastric varices or those who experienced acute bleeding related to esophageal varices. Despite this, their cirrhosis-related complications were not severe (without hepatorenal syndrome, hepatic encephalopathy, and spontaneous bacterial peritonitis). Exclusion criteria were as follows: (1) patients with hepatocellular carcinoma (HCC); (2) patients treated with splenectomy; (3) patients treated with transjugular intrahepatic portosystemicstent-shunt (TIPS); (4) patients treated with subtotal gastrectomy; and (5) patients taking propranolol or other NSBBs. Clinical data (including age, gender, underlying disease, etiology, ALBI stage, ascites, Child-Pugh stage, child-turcotte-pugh (CTP) classification, a diameter of esophageal varices, presence of gastric varices, prophylactic or acute bleeding, and whether the gastric varices were injected with tissue adhesives) and laboratory data [ammonia, white blood cells (WBC). Hemoglobin (Hb), platelets (PLTs), alanine transaminase (ALT), aspartate transaminase (AST), γ-glutamyl transpeptidase (GGT), cholinesterase (CHE), international standard ratio (INR), creatinine (CR), urea nitrogen (Urea), glucose (GLU), total bilirubin (TBIL), albumin (ALB)] at the initial treatment were collected before. Medical history was also collected, including hypertension, diabetes, and coronary heart disease. The Ethics Committee approved this retrospective cohort study of Tianjin Second People's Hospital, and written consent was obtained from the patients before the study.

### Endoscopic treatment

EVL was performed using a multi-band ligature (T.Y. Medical organism material research Co., Ltd., Tianjin, China). Variceal ligation was performed starting from the nearest position near the gastroesophageal junction, releasing five to seven bands depending on the variceal condition.

**Figure 1:**
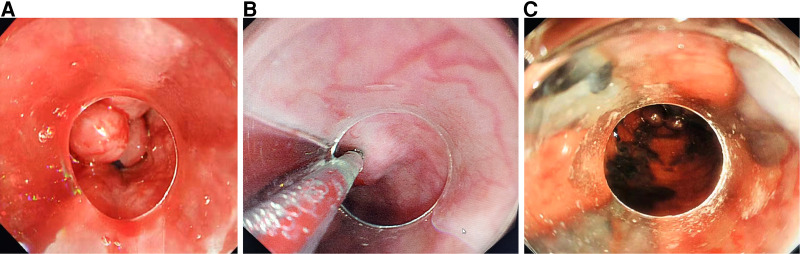
(**A**) Esophageal variceal ligation, (**B**) sclerosant injecting into each varicose vein, and (**C**) sclerosant in the varicose veins.

For patients receiving EVL plus EIS, EVL was performed first, in the same way as in the EVL group. Then a puncture needle (23-gauge) was inserted into varicosity 1–2 cm proximal to the upper part of the band and injected with lauromacrogol (Tianyu Pharmaceutical, Shanxi, China) on the same day, 2–8 ml at a time, as a sclerosant (Figure 1).

**Figure 2:**
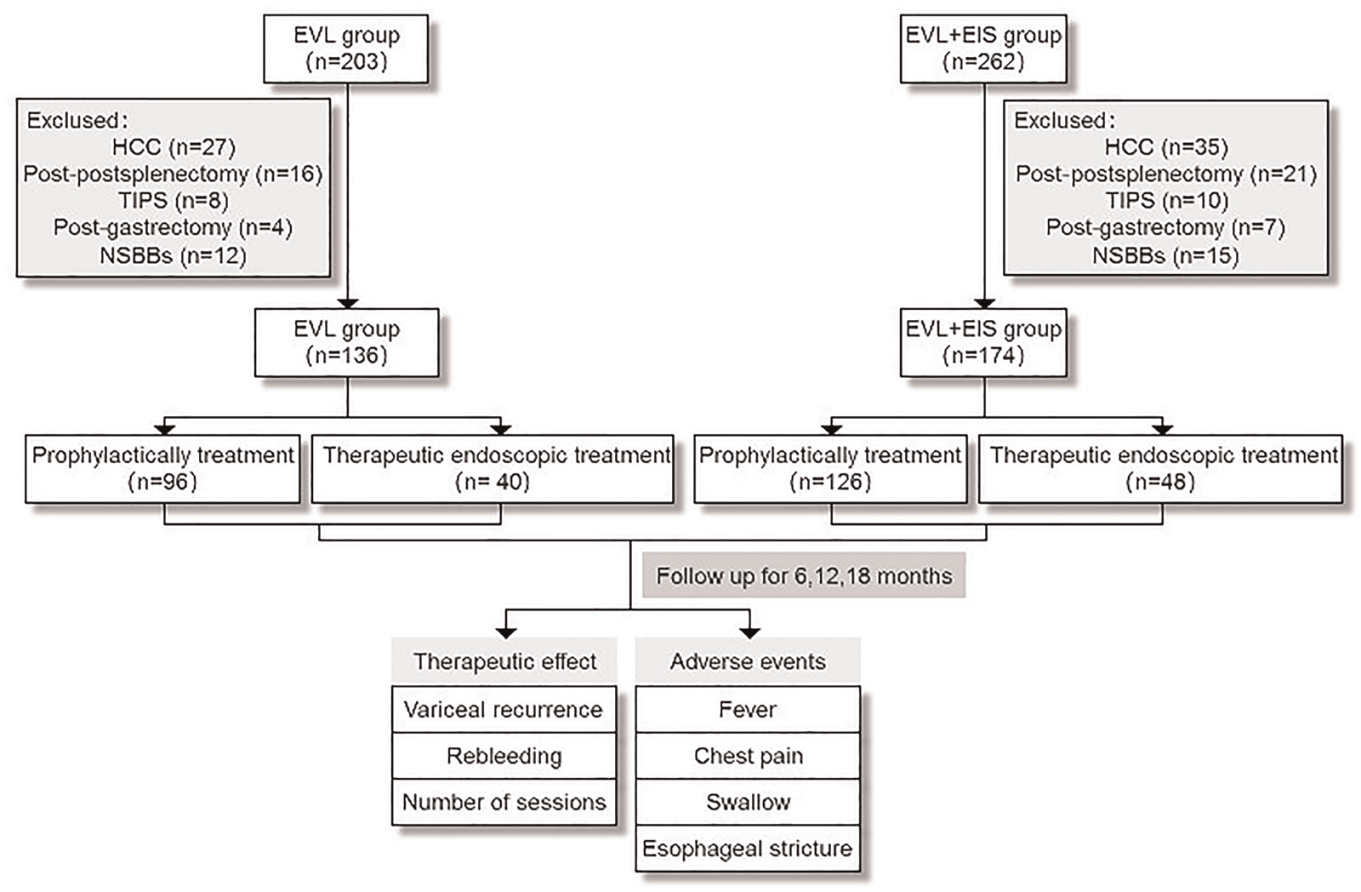
Flow diagram of this study population. EVL, esophageal variceal ligation; EIS, endoscopic injection sclerosis; HCC, hepatocellular carcinoma; TIPS, transjugular intrahepatic portosystemicstent-shunt; NSBBs, non-selective beta-blockers.

### Follow-up and outcome assessment

After the initial treatment, the treated esophageal varices are examined endoscopically 2 or 3 weeks later. Repeated treatments were then performed until the esophageal varices were eradicated. The primary follow-up outcome was the number of sessions until eradication of esophageal varices and rebleeding and variceal recurrence assessed at 6, 12, and 18 months after the eradication of esophageal varices. Rebleeding was defined as clinical hematemesis or melena which was confirmed by endoscopy as esophagogastric varices bleeding. Variceal recurrence was defined as the presence of varicose veins requiring retreatment. The number of sessions was defined as the number of sessions performed to eradicate the varicose veins.

### Study outcomes

For the first objective, the primary outcomes were efficacy and adverse events between control and intervention groups. For the second objective, we further estimated the efficacy difference between prophylactic and therapeutic endoscopic treatment patients in the two groups.

### Statistical analysis

In the case of normal distribution, the data were presented as mean ± standard deviation, or amount and percentage. Predictors of varicose vein recurrence were identified by logistic regression analysis. Propensity scores were selected from propensity score matching (PSM) analysis to overcome the bias introduced by the different distributions in each group. All data were analyzed by R software v3.6.1 (R Foundation for Statistical Computing, Vienna, Austria). The “Matchlt” package was used in the PSM matching. Comparisons of baseline parameters and outcomes between the two groups were performed using Student's *t* test and *χ*^2^ test. *P*-values <0.05 were considered statistically significant.

## Results

### Patient characteristics

A total of 310 patients in the control group (*n* = 136) and intervention group (*n* = 174) participated in this study after a 6-month follow-up for varicose vein recurrence, rebleeding rates, number of sessions, and adverse events. Of these patients, 252 patients (control group, *n* = 136 and intervention group, *n* = 116) completed the 12-month follow-up and 206 patients (control group, *n* = 136 and intervention group, *n* = 70) completed the 18-month follow-up. Before PSM matching, there were significant gender differences, esophageal variceal diameter, tissue adhesive injection or not, INR, ammonia, ascites, and CTP classification between the control and intervention groups at 12-month follow-up (*P *< 0.05). Table 1 summarizes the characteristics of the two cohorts. At 6-month follow-up patients, there were significant differences between the control and intervention groups regarding gender, esophageal variceal diameter, gastric varices, tissue adhesive injection, and CTP classification (*P *< 0.05). Among patients at 18-month follow-up, the proportion of men was significantly higher in the intervention group than in the control group before PSM (*P* = 0.006). At the same time, the esophageal variceal diameter was significantly larger in the intervention group (*P* < 0.001). Statistically, the intervention group had a statistically significant proportion of tissue adhesive injection (*P* < 0.001), a higher INR (*P* < 0.001), and higher ammonia (*P* = 0.001). Statistically significant findings were also found for ascites and CTP classification in the control and intervention groups (*P* = 0.016 and *P* = 0.031). After PSM, there were no differences between the two groups in terms of gender, esophageal variceal diameter, tissue adhesive injection, INR, ascites, and CTP classification (all *P* > 0.05) (Table 1).

**Table 1:** Baseline patient characteristics.

	Before PSM			After PSM		
	EVL	EVL + EIS	*P*	EVL	EVL + EIS	*P*
	*n* = 136	*n* = 116		*n* = 69	*n* = 69	
Gender, male	76 (55.9)	85 (73.3)	0.006	47 (68.1)	49 (71.0)	0.853
Age, year	53.29 ± 10.74	53.60 ± 9.97	0.81	52.86 ± 11.28	53.52 ± 9.18	0.704
Hypertension, yes	27 (19.9)	20 (17.2)	0.713	14 (20.3)	14 (20.3)	1
Diabetes, yes	30 (22.1)	25 (21.6)	1	16 (23.2)	17 (24.6)	1
CHD, yes	15 (11.0)	7 (6.0)	0.24	9 (13.0)	2 (2.9)	0.059
Etiology						
Hepatitis B	83 (61.0)	71 (61.2)	0.985	41 (59.4)	44 (63.8)	0.156
Hepatitis C	15 (11.0)	13 (11.2)		10 (14.5)	7 (10.1)	
Autoimmune	9 (6.6)	6 (5.2)		1 (1.4)	5 (7.2)	
Alcoholic	11 (8.1)	11 (9.5)		3 (4.3)	6 (8.7)	
Other	18 (13.2)	15 (12.9)		14 (20.3)	7 (10.1)	
Child-pugh stage						
A	72 (52.9)	73 (62.9)	0.277	41 (59.4)	40 (58.0)	0.899
B	57 (41.9)	38 (32.8)		26 (37.7)	26 (37.7)	
C	7 (5.1)	5 (4.3)		2 (2.9)	3 (4.3)	
Treatmint prupose						
Primary prophylaxis	64 (47.1)	56 (48.3)	0.974	31 (44.9)	39 (56.5)	0.393
Secondary prophylaxis	32 (23.5)	26 (22.4)		17 (24.6)	13 (18.8)	
Acute variceal bleeding	40 (29.4)	34 (29.3)		21 (30.4)	17 (24.6)	
Diameter of esophageal varices, cm	0.92 ± 0.18	1.09 ± 0.20	<0.001	0.99 ± 0.13	1.04 ± 0.18	0.067
Gastric varices						
None + mild	36 (26.5)	39 (33.6)	0.272	25 (36.2)	26 (37.7)	1
Moderate + severe	100 (73.5)	77 (66.4)		44 (63.8)	43 (62.3)	
Tissue adhesive injection, yes	13 (9.6)	41 (35.3)	<0.001	10 (14.5)	20 (29.0)	0.063
WBC, 10^9^/L	3.94 ± 2.06	3.90 ± 1.74	0.848	3.94 ± 2.12	3.89 ± 1.81	0.895
Hb, g/L	104.34 ± 26.01	101.75 ± 29.59	0.461	100.68 ± 25.71	103.71 ± 28.24	0.511
PLT, 10^9^/L	77.56 ± 44.65	83.09 ± 65.99	0.431	79.48 ± 45.48	74.00 ± 47.97	0.492
ALT, U/L	31.34 ± 39.00	25.31 ± 23.43	0.147	29.45 ± 40.80	28.64 ± 28.95	0.893
AST, U/L	42.57 ± 47.80	34.63 ± 30.16	0.124	36.54 ± 26.59	37.62 ± 34.48	0.836
GGT, U/L	79.05 ± 106.94	61.73 ± 72.09	0.14	75.65 ± 98.64	66.30 ± 78.16	0.538
CHE, U/L	4,126.90 ± 1,434.64	4,148.27 ± 1,477.65	0.908	4,132.86 ± 1,492.19	4,186.13 ± 1,609.67	0.841
INR	1.34 ± 0.28	1.22 ± 0.17	<0.001	1.29 ± 0.22	1.25 ± 0.17	0.241
CR, µmol/L	62.67 ± 21.77	67.25 ± 42.96	0.276	64.67 ± 21.96	64.54 ± 18.12	0.97
Urea, mmol/L	6.29 ± 2.96	5.98 ± 3.04	0.413	6.15 ± 2.86	5.51 ± 2.60	0.175
GLU, mmol/L	7.00 ± 2.48	6.62 ± 1.69	0.158	7.02 ± 2.81	6.58 ± 1.52	0.254
Ammonia, µmol/L	29.32 ± 18.63	37.07 ± 17.25	0.001	35.39 ± 19.87	36.16 ± 17.85	0.811
TBIL, µmol/L	29.52 ± 40.52	27.24 ± 27.71	0.61	28.18 ± 37.39	27.54 ± 19.51	0.899
ALB, g/L	36.40 ± 6.11	35.70 ± 5.68	0.346	36.32 ± 6.35	36.06 ± 5.54	0.802
Ascites						
None	62 (45.6)	75 (64.7)	0.016	40 (58.0)	41 (59.4)	0.878
Mild	36 (26.5)	23 (19.8)		19 (27.5)	17 (24.6)	
Moderate	28 (20.6)	15 (12.9)		8 (11.6)	10 (14.5)	
Severe	10 (7.4)	3 (2.6)		2 (2.9)	1 (1.4)	
MELD score	60.15 ± 5.14	59.65 ± 5.11	0.436	59.96 ± 4.63	60.15 ± 4.27	0.808
ALBI stage						
1	31 (22.8)	25 (21.6)	0.808	17 (24.6)	15 (21.7)	0.848
2	93 (68.4)	83 (71.6)		47 (68.1)	50 (72.5)	
3	12 (8.8)	8 (6.9)		5 (7.2)	4 (5.8)	
CTP classification						
A	62 (45.6)	72 (62.1)	0.031	39 (56.5)	39 (56.5)	0.922
B	62 (45.6)	38 (32.8)		27 (39.1)	26 (37.7)	
C	12 (8.8)	6 (5.2)		3 (4.3)	4 (5.8)	

Data are given as mean ± SD, or *n* (%).

PSM, propensity score matching; EVL, esophageal variceal ligation; EIS, endoscopic injection sclerosis; CHD, coronary heart disease; WBC, white blood cell; Hb, hemoglobin; PLT, platelet; ALT, alanine transaminase; AST, aspartate transaminase; GGT, γ-glutamyl transpeptadase; CHE, cholinesterase; INR, international standard ratio; CR, creatinine; Urea, urea nitrogen; GLU, glucose; TBIL, total bilirubin; ALB, albumin; MELD, model for end-stage liver disease.

### Primary outcomes

At 12-month follow-up, rebleeding occurred in 3 patients (2.6%) in the intervention group, much lower than in 14 patients (10.3%) in the control group (*P *= 0.006). The rate of variceal recurrence was lower in the intervention group (24 patients, 20.7%) than in the control group (51 patients, 37.5%) (*P* = 0.029). The mean number of sessions in the control group were 31 for one time, 55 for two times, 44 for three times, and 6 for four times. However, the number of sessions were 55 for one time, 34 for two times, 24 for three times, and 3 for four times in the intervention group (*P* = 0.001). After PSM-matched analysis, two patients (2.9%) in the intervention group had rebleeding, similar to the control group (*P* = 0.101). Variceal recurrence occurred in 12 (17.4%) patients in the intervention group and 27 (39.1%) patients in the control group (*P* = 0.008). Of the patients in the intervention group, 37 were treated one time, 19 for two times, 13 for three times, and 0 for four times. Fifteen for one time, 31 for two times, 19 for three times, and 4 for four times in the control group (*P *= 0.001). There was also no significant difference in rebleeding rates between control and intervention groups after PSM matching patients at 6-month follow-up (*P* = 0.612). Similarly, the rate of variceal recurrence proved to be equal in both matched groups (*P* = 0.076). Table 2 shows all comparisons of varicose recurrence, rebleeding, and the number of sessions between these two groups at 6, 12, and 18 months.

**Table 2:** Therapeutic efficacy.

		6 months			12 months			18 months		
		EVL	EVL + EIS	*P*	EVL	EVL + EIS	*P*	EVL	EVL + EIS	*P*
		*n* = 136	*n* = 174		*n* = 136	*n* = 116		*n* = 136	*n* = 70	
Before PSM	Variceal recurrence, yes	32	19	0.005	51	24	0.006	64	18	0.005
	Rebleeding, yes	8	2	0.044	14	3	0.029	16	1	0.022
	Number of sessions,									
	1/2/3/4	31/55/44/6	89/52/30/3	<0.001	31/55/44/6	55/34/24/3	0.001	31/55/44/6	36/20/12/2	0.001
		EVL	EVL + EIS	*P*	EVL	EVL + EIS	*P*	EVL	EVL + EIS	*P*
		*n* = 77	*n* = 77		*n* = 69	*n* = 69		*n* = 54	*n* = 54	
After PSM	Variceal recurrence, yes	16	6	0.038	27	12	0.008	26	16	0.076
	Rebleeding, yes	3	1	0.612	8	2	0.101	8	1	0.037
	Number of sessions,									
	1/2/3/4	16/35/22/4	39/21/16/1	0.001	15/31/19/4	37/19/13/0	0.001	11/26/14/3	26/17/10/1	0.022

EVL, esophageal variceal ligation; EIS, endoscopic injection sclerosis; PSM, propensity score matching.

### Secondary outcomes

We analyzed prophylactic and therapeutic endoscopic treatments separately. At the 12-month follow-up, the incidence of fever, chest pain, or swallowing did not differ significantly between the control and intervention groups. There were no significant differences in rebleeding, variceal recurrence, or the number of sessions for patients treated with therapeutic endoscopy between the control and intervention groups. However, the rate of rebleeding was lower in the intervention group (1, 1.2%) than in the control group (9, 9.4%) (*P* = 0.042); 14 (17.1%) patients in the intervention group experienced variceal recurrence, much lower than 33 (34.4%) patients in the control group (*P *= 0.015) during prop hylactically treatment. The number of sessions in the control group compared with the intervention group was significantly different (*P *< 0.001) (Table 3).

**Table 3:** Prophylactically and therapeutically endoscopic treatments in 12 months.

		Prophylactically treatment			Therapeutically treatment		
		EVL	EVL + EIS	*P*	EVL	EVL + EIS	*P*
		*n* = 136	*n* = 174		*n* = 136	*n* = 174	
Variceal recurrence (%)	No	63 (65.6)	68 (82.9)	0.015	22 (55.0)	24 (70.6)	0.255
	Yes	33 (34.4)	14 (17.1)		18 (45.0)	10 (29.4)	
Rebleeding (%)	No	87 (90.6)	81 (98.8)	0.042	35 (87.5)	32 (94.1)	0.568
	Yes	9 (9.4)	1 (1.2)		5 (12.5)	2 (5.9)	
Number of sessions (%)	1	22 (22.9)	44 (53.7)	<0.001	9 (22.5)	11 (32.4)	0.629
	2	38 (39.6)	23 (28.0)		17 (42.5)	11 (32.4)	
	3	31 (32.3)	14 (17.1)		13 (32.5)	10 (29.4)	
	4	5 (5.2)	1 (1.2)		1 (2.5)	2 (5.9)	
Fever (%)	No	85 (88.5)	76 (92.7)	0.496	39 (97.5)	32 (94.1)	0.886
	Yes	11 (11.5)	6 (7.3)		1 (2.5)	2 (5.9)	
Chest pain (%)	No	74 (77.1)	62 (75.6)	0.957	28 (70.0)	28 (82.4)	0.336
	Yes	22 (22.9)	20 (24.4)		12 (30.0)	6 (17.6)	
Swallow (%)	No	86 (89.6)	71 (86.6)	0.7	35 (87.5)	31 (91.2)	0.895
	Yes	10 (10.4)	11 (13.4)		5 (12.5)	3 (8.8)	

EVL, esophageal variceal ligation; EIS, endoscopic injection sclerosis.

### Development of predictive factors

Univariate logistic regression analysis was performed on the intervention group and other clinical or demographic characteristics to develop predictors of variceal recurrence in patients at 12-month follow-up. Univariate logistic regression analysis showed that esophageal variceal diameter [odds ratio (OR) = 15.856; 95% confidence interval (CI), 1.709–160.143; *P* = 0.016] and gastric varices (OR = 4.5; 95% CI, 1.42–20.028; *P* = 0.021) were statistically associated with variceal recurrence in patients who received EVL plus EIS. Meanwhile, based on multivariate logistic regression analysis, the diameter of esophageal varices (OR = 10.673; 95% CI, 1.051–116.294; *P* = 0.047) and gastric varices (OR = 3.795; 95% CI, 1.163–17.128; *P* = 0.045) were shown to be predictors of variceal recurrence in patients in the intervention group (Table 4).

**Table 4:** Logistic regression analysis of intervention group.

		EVL + EIS					
		Unitivariate logistic regression			Multivariate logistic regression		
		OR	95% CI	*P*	OR	95% CI	*P*
Etiology	Hepatitis B	Reference					
	Hepatitis C	1.12	0.23–4.226	0.875			
	Autoimmune	0.747	0.037–5.111	0.797			
	Alcoholic	0.83	0.118–3.665	0.823			
	Other	0.933	0.195–3.413	0.922			
Child-pugh stage	A	Reference					
	B	0.804	0.283–2.105	0.667			
	C	0.891	0.044–6.564	0.92			
Ascites	None	Reference					
	Mild	1.21	0.353–3.66	0.745			
	Moderate	1.089	0.227–4.009	0.904			
	Severe	8.714	0.783–195.496	0.086			
ALBI stage	1	Reference					
	2	1.559	0.514–5.829	0.463			
	3	0.75	0.035–6.234	0.811			
CTP classification	A	Reference					
	B	0.79	0.278–2.069	0.642			
	C	0.7	0.035–4.767	0.753			
Treatment purpose	Primary prophylaxis	Reference					
	Secondary prophylaxis	0.836	0.211–2.815	0.782			
	Acute variceal bleeding	1.917	0.696–5.308	0.205			
Age		1.028	0.982–1.079	0.256			
ALB		1.004	0.926–1.087	0.931			
ALT		0.97	0.922–1.003	0.185			
Ammonia		0.991	0.964–1.017	0.507			
AST		0.98	0.945–1.003	0.2			
CHE		1	1–1	0.703			
CR		0.997	0.976–1.008	0.686			
Diameter of esophageal varices		15.856	1.709–160.143	0.016	10.673	1.051–116.294	0.047
Gastric varices		4.5	1.42–20.028	0.021	3.795	1.163–17.128	0.045
Gender		0.524	0.203–1.398	0.185			
GGT		0.994	0.982–1.002	0.276			
GLU		0.883	0.64–1.161	0.406			
Tissue adhesive injection		2.172	0.868–5.469	0.096			
Hb		0.994	0.979–1.009	0.452			
INR		0.512	0.027–7.668	0.639			
MELD		0.932	0.847–1.02	0.136			
PLT		1.002	0.995–1.008	0.573			
TBIL		0.965	0.92–0.998	0.086			
Urea		1.015	0.867–1.167	0.845			
WBC		1.007	0.768–1.297	0.96			

OR, odds ratio; WBC, white blood cell; Hb, hemoglobin; PLT, platelet; ALT, alanine transaminase; AST, aspartate transaminase; GGT, γ-glutamyl transpeptadase; CHE, cholinesterase; INR, international standard ratio; CR, creatinine; Urea, Urea nitrogen; GLU, glucose; TBIL, total bilirubin; ALB, albumin; MELD, model for end-stage liver disease.

### Adverse events after therapy

In the 12-month follow-up patients, the incidence rate of fever (*P* = 1), chest pain (*P* = 0.676), and swallowing (*P* = 0.79) in the intervention group were similar to those of patients in the control group. In all patients, there were no patients with esophageal stricture. After the PSM matching analysis, the adverse events (fever, chest pain, swallowing, and esophageal stricture) also showed no significant difference between the intervention group and control groups. For the patients after 6 months and 12-month follow-up before and after the PSM matching, the incidence rate of adverse events was also similar between the two groups (Table 5).

**Table 5:** Adverse events.

	6 months			12 months			18 months			
		EVL	EVL + EIS	*P*	EVL	EVL + EIS	*P*	EVL	EVL + EIS	*P*
	*n* = 136	*n* = 174	* *	*n* = 136	*n* = 116	* *	*n* = 136	*n* = 70	* *	
Before PSM	Fever, yes	12	15	1	12	8	0.741	12	3	0.366
	Chest pain, yes	34	42	0.966	34	26	0.74	34	14	0.529
	Swallow, yes	15	20	1	15	14	0.952	15	9	0.874
	Esophageal stricture, yes	0	0	NA	0	0	NA	0	0	NA
		EVL	EVL + EIS	*P*	EVL	EVL + EIS	*P*	EVL	EVL + EIS	*P*
		*n* = 77	*n* = 77		*n* = 69	*n* = 69		*n* = 54	*n* = 54	
After PSM	Fever, yes	7	6	1	4	5	1	3	2	1
	Chest pain, yes	17	12	0.41	16	13	0.676	12	13	1
	Swallow, yes	9	2	0.06	9	7	0.79	4	6	0.74
	Esophageal stricture, yes	0	0	NA	0	0	NA	0	0	NA

EVL, esophageal variceal ligation; EIS, endoscopic injection sclerosis; PSM, propensity score matching.

## Discussion

In our study, we observed the variceal recurrence and rebleeding after the eradication of esophageal varicose for the follow-up of 6 months, 12 months, and 18 months, respectively. Because many clinical factors influence variceal recurrence and rebleeding, we balanced the baseline clinical data by PSM. Prior to PSM, our study showed that patients treated with EVL plus EIS had significantly less varicose recurrence and rebleeding than patients treated with EVL alone, whether at 6-, 12-, or 18-month follow-up. Our results agree with Mansour et al. and Wang et al. (2, 10, 14, 15). Compared with EVL alone, the EVL plus EIS group had a significant advantage in variceal recurrence and rebleeding for the following principal reasons. (1) Many patients have disappeared distal esophageal varices after EVL, but the upper segment veins are still prominent. Therefore, our approach allows injection of the sclerosing agent into the upper varicose vein and diffuses in the interconnected perforating vessels or venous blood supply, preventing variceal recurrence and rebleed. (2) EVL plus EIS allows the sclerosing agent to remain in the vein for a more extended period after the ligature has partially blocked the blood flow, thus producing more chemical effects on the venous endothelium. To make the results more credible, we used the method of PSM. After PSM, for variceal recurrence, the EVL plus EIS group remained significantly lower than the EVL group at the 6- and 12-month follow-ups. However, there was no significant difference between the two groups at the 18-month follow-up. This may be due to the persistence of portal hypertension, where a new one inevitably replaces the disappearing collateral circulation. Rebleeding was also more frequent in the EVL group than in the EVL plus EIS group after PSM; however, the difference was not statistically significant at the 6- and 12-month follow-ups, which was different from the results before PSM but similar to those of Mansour et al. (14). At the 18-month follow-up, for rebleeding, the EVL plus EIS group was significantly lower than the EVL group. The possible reasons for this are unclear and need further validation by expanding the sample size. Our study also showed that the number of sessions performed for complete eradication of varices in the EVL plus EIS group was significantly less than in the EVL alone group similar to the study conducted by Mansour et al. (14). The advantage of fewer sessions with fewer hospitalizations and costs. Interestingly, we also found that the EVL plus EIS group had less variceal obliteration and fewer variceal recurrences and rebleeds in prophylactic-treated patients at 12-month follow-up; however, for prophylactic-treated patients, this was meaningful. However, there was no statistical significance for patients with acute bleeding, which may be related to poor visualization and overall poor status. Therefore, EVL or EVL plus EIS is possible for patients with acute bleeding, but for prophylaxis, the combination is more advantageous, which has not been reported. In previous studies, 2%–20% of patients experienced adverse events of EVL, including bleeding after taping, transient dysphagia, retrosternal pain, esophageal ulcer, esophageal stricture, and esophageal perforation. Many patients develop adverse events of sclerotherapy, including fever, dysphagia, esophageal stricture, retrosternal discomfort, esophageal ulceration with bleeding, pneumothorax, and esophageal perforation, pleural effusion, and mediastinitis (16, 17). In our study, patients in both groups did not experience any fatal adverse events. Neither group did not have esophageal perforation, pleural effusion, pneumothorax, or mediastinitis. Three cases in the EVL group developed esophageal ulceration with bleeding within 6 weeks of treatment and subsequently received TIPS. This did not occur in the EVL plus EIS group. The two groups had no significant differences in adverse events such as fever, chest pain, swallowing, and esophageal stricture. These results are consistent with previous studies (2, 10, 14, 15). Possible reasons for this result are as follows: (1) regular use of PPI reduces the risk of ligation-induced ulceration; (2) treatment by experienced physicians; (3) injection of only sclerosant for esophageal varices, not tissue adhesives; and (4) precise intravascular injections at a slower rate.

EVL is the recommended option for treating esophageal varices and esophageal variceal bleeding (18). However, EVL requires the placement of a cylinder in front of the endoscope, which affects the endoscopic view. EVL is not suitable for varices larger than 2 cm or smaller than 0.5 cm in diameter. EIS is limited mainly due to its adverse events. Some investigators believe that EVL combined with EIS can eradicate superficial varices in the esophageal mucosa and block deeper traffic branches and small varices in the esophageal wall. In addition, using EVL may reduce the required dose of lauromacrogol, reducing the risk of adverse events (19, 20). Several recent studies have used EVL and EIS sequentially or in combination to treat esophageal varices with significant results. Sang et al. and Zhou et al. performed sequential sclerotherapy after ligation (21, 22). Tajiri et al. described the treatment of esophageal varices with injectable sclerotherapy followed by ligation (23). Mansour et al. and Harras et al. performed sclerotherapy at each variceal vein 3–5 cm and 5–10 cm from the esophagogastric junction and then injected sclerosing agent into the variceal vein 2–3 cm above the gastroesophageal junction (14, 24). Wang et al. ligated each vein and then injected 2–5 ml of a sclerosing agent into the variceal vein at the time of ligation, 2–3 cm from the upper part of the band (10). In our study, we first performed EVL at the nearest location near the gastroesophageal junction, releasing 5–7 bands depending on the varices to maximize blockage of blood flow. Then injected 2–8 ml of the lauromacrogol in the varices simultaneously as sclerosis, 1–2 cm from the upper part of the band. The advantages of our procedure are as follows: (1) the transparent cap is still present at the tip of the lens after EVL, which can fix the varicose vein and facilitate sclerotherapy and can also be used for compression if there is leakage; (2) performing EVL followed by EIS can also reduce the risk of bleeding after ligation of the thick varicose vein; (3) performing EVL earlier can reduce the pressure in the varicose vein and create conditions for subsequent sclerotherapy (less sclerosing agent, long sclerosing agent dwell time); and (4) the procedure does not require special skills and equipment. But, at present, there are still controversies about the efficacy and adverse events of EVL plus EIS and EVL alone for esophageal varicose (2, 10, 14, 15). A meta-analysis discovered that EVL is superior to the combination of EVL and EIS in safety, while no significant differences were found in efficacy (2). However, some studies support that the combination of EVL and EIS is better than EVL alone (10, 14, 15). For example, Jianbo Wang et al. retrospectively analyzed the rebleeding rate and variceal recurrence at 6 months in 84 patients, of which 40 patients were treated with EVL alone and 44 patients were treated with EVL plus EIS and indicated that EVL plus EIS showed less rebleeding rates and variceal recurrence at 6 months and less chest pain and was more cost-effective compared to EVL alone in the treatment of gastroesophageal varices. In addition, a network meta-analysis also reported that EVL combined with EIS might be the most efficacious intervention for preventing rebleed, mortality, and bleeding-related death (25). Compared with previous research, our study has a larger sample size and longer follow-up time. In addition, PSM was performed to balance the biases of these two groups. If the effect of the intervention group is effective then the varices should be eradicated in fewer sessions and the variceal recurrence should be lower, and the adverse events should be equal to or even lower than EVL alone.

Our study also has certain limitations. It was a single-center retrospective study. The regression analysis did not include more clinical parameters, and long-term follow-up of patients' deaths was impossible. Further large-scale studies need to be organized in the future.

## Conclusion

EVL plus EIS has lower variceal recurrence and rebleeding, compared with EVL alone, and has no obvious adverse events in our study. But we still need prospective, multicenter, randomized controlled studies to further confirm, in order to provide better treatment for esophageal varices.

## Data Availability

The original contributions presented in the study are included in the article/Supplementary Material, further inquiries can be directed to the corresponding authors.
